# GABr Post-Treatment for High-Performance MAPbI_3_ Solar Cells on Rigid Glass and Flexible Substrate

**DOI:** 10.3390/nano11030750

**Published:** 2021-03-16

**Authors:** Tingting Chen, Rui He, Fan Zhang, Xia Hao, Zhipeng Xuan, Yunfan Wang, Wenwu Wang, Dewei Zhao, Jingquan Zhang, Lili Wu

**Affiliations:** 1 College of Materials Science and Engineering, Sichuan University, Chengdu 610065, China; chentingting1@stu.scu.edu.cn (T.C.); hrkbjj@stu.scu.edu.cn (R.H.); www1492@scu.edu.cn (W.W.); dewei.zhao@scu.edu.cn (D.Z.); zhangjq@scu.edu.cn (J.Z.); 2 Institute of New Energy and Low-Carbon Technology, Sichuan University, Chengdu 610065, China; zhangfan3@stu.scu.edu.cn (F.Z.); 2018226220003@stu.scu.edu.cn (Z.X.); wangyunfan@stu.scu.edu.cn (Y.W.); 3 Engineering Research Center of Alternative Energy Materials & Devices, Ministry of Education, Chengdu 610065, China

**Keywords:** nonradiative recombination, post-treatment, flexible perovskite solar cell

## Abstract

Perovskite solar cells have exhibited astonishing photoelectric conversion efficiency and have shown a promising future owing to the tunable content and outstanding optoelectrical property of hybrid perovskite. However, the devices with planar architecture still suffer from huge *V*_oc_ loss and severe hysteresis effect. In this research, Guanidine hydrobromide (GABr) post-treatment is carried out to enhance the performance of MAPbI_3_ n-i-p planar perovskite solar cells. The detailed characterization of perovskite suggests that GABr post-treatment results in a smoother absorber layer, an obvious reduction of trap states and optimized energy level alignment. By utilizing GABr post-treatment, the *V*_oc_ loss is reduced, and the hysteresis effect is alleviated effectively in MAPbI_3_ solar cells. As a result, solar cells based on glass substrate with efficiency exceeding 20%, *V*_oc_ of 1.13 V and significantly mitigated hysteresis are fabricated successfully. Significantly, we also demonstrate the effectiveness of GABr post-treatment in flexible device, whose efficiency is enhanced from 15.77% to 17.57% mainly due to the elimination of *V*_oc_ loss.

## 1. Introduction

Organic–inorganic hybrid perovskites have received great attention due to their superior properties of high light absorption coefficient [1], low exciton binding energy [2], and long diffusion length [3], which are suitable for application in photovoltaic devices. Lead-based organic perovskite (APbX_3_, A = CH_3_NH_3_^+^, CH(NH_2_)_2_^+^, alkali metal ion or mixed cations; X = I, Cl, Br or mixed anions) is easy to fabricate and the power conversion efficiency (PCE) for the state-of-the-art device has achieved 25.5% [4]. Meanwhile, the stability obstacle is gradually being overcome after numerous efforts have been paid to it [5,6]. MAPbI_3_ is the commonly used material in perovskite solar cell (PSCs) with lower Urbach Energy (~19 meV) than other photovoltaic materials and is relatively cheap to produce with simple composite [7]. The development of MAPbI_3_-based PSCs is rapid, but the device performance still suffers from nonnegligible nonradiative recombination [8,9]. MAPbI_3_ is compatible with various fabrication techniques, and among which the solution process is commonly taken in experiments due to the advantage of low temperature and easy operation. However, the film deposited through solution method is usually polycrystalline with high density of defects situated at the grain boundary or the film surface, which act as nonradiative recombination centers and result in serious efficiency loss [10,11,12,13,14]. Besides, the existence of defects will induce ion migration in perovskite layer and thus cause serious hysteresis in solar cells which brings difficulty in determining the accurate efficiency of PSCs [15,16].

Compositional engineering and surface modification are two main strategies to improve the performance of solar cells. Guanidinium cation (GA^+^) is an effective defect passivator due to the unique hydrogen bonding capability [17]. In 2014, GASCN and GANO_3_ were employed as additives in dye-sensitized solar cells to decrease the electron/iodine recombination and enhance the collection efficiency [18]. Yang et al. doped GAI into MAPbI_3_ precursor directly and promoted the PCE to over 17%. They found out that the incorporation of GA could improve the film continuity without changing the crystal structure of perovskite [19]. Zhou et al. introduced GABr into FA_0.7_MA_0.3_Pb_0.7_Sn_0.3_I_3_ perovskite and found that GABr could reduce the defects caused by the oxidation of Sn^2+^ and hence improve the PCE and stability performance [20]. Except for introducing GA^+^ into perovskite composition, several studies employed GA salts treatment on perovskite layer and the performance optimization of devices were also observed [21,22,23]. Yan et al. developed CsPbI_3_ PSCs through GABr treatment and they proposed that this treatment would induce ion exchange between I^−^ and Br^−^ and then cause the formation of 2D GA-CsPbI_x_Br_y_ to passivate defects [21]. In Chen’s work, they applied a GABr solution to modify the wide-bandgap perovskite surface and demonstrated the formation of a graded perovskite homojunction which resulted in effective grain boundary passivation after treatment [22]. In Zhang’s research, GABr post-treatment induced a 2D/3D heterostructure in Cs_(1−x)_Rb_x_PbI_2_Br perovskite and reduced the density of defects [23].

Except for energy loss caused by defects state, MAPbI_3_ n-i-p planar solar cell suffers from a severe hysteresis effect. The rapid nucleation and grain growth might be the main reasons for the formationof defective films and the hysteresis is suspected to be caused by defect induced ion migration, the fundamental properties of MAPbI_3_, e.g., the dipole moment, and so on [24]. Therefore, GABr treatment on MAPbI_3_ seems to be ideal in optimizing the device performance for the two reasons: (1) the nearly zero dipole moment of GA^+^ is beneficial for adjusting the polarity for perovskite to reduce the hysteresis [25,26], (2) Br^−^ will compete with I^−^ in I-based perovskite and can retard the crystal process to achieve less-defective films [20,27]. However, the application of GABr post-treatment on MAPbI_3_ PSCs with n-i-p planar structure has been rarely reported and it is worth exploring.

Thus, we attempt to utilize GABr post-treatment to modify the MAPbI_3_ PSCs with n-i-p planar architecture. Notably, if the content of GABr is too high, the phase segregation might be induced [28] and the large GA cation will block the charge transfer which can result in poor device performance [29,30]. Therefore, special care should be taken while the GABr treatment is carried out.

Among the lead-based organic perovskites, MAPbI_3_-based planar PSCs is one especially appropriate architecture to develop flexible photovoltaic cell as the annealing temperature is compatible with the plastic substrates. It is well known that flexible cells have been rapidly developed due to their light weigh, excellent bending resistance, and suitablility for being employed in wearable electronics, flying objects, and smart textiles. However, the development of flexible PSCs still suffers from poor efficiency which is much lower than rigid PSCs. To the best of our knowledge, the state-of-the-art PCE of rigid MAPbI_3_-based PSCs has reached 21.88% [31] and several works have been carried out to improve the efficiency of flexible PSCs to over 18% [8,32,33,34]. Considering that the low-temperature manufacturing of GABr treatment (<150 °C) and the previous applications in improving the device performance, it is expected to utilize this optimization method to improve the efficiency of flexible devices [21,22,35,36].

In this work, we conducted a simple GABr post-treatment and investigated the effects on MAPbI_3_ films and solar cells. Thanks to the improved film quality, reduced defect states, and suppressed hysteresis, an optimized performance is demonstrated in both rigid and flexible MAPbI_3_ based PSCs.

## 2. Materials and Methods

### 2.1. Materials

The indium tin oxide (ITO) glass substrate was purchased from Pilkington Group Limited, and the PEN-ITO substrate was purchased from Peccell (Kanagawa, Japan). Lead (II) iodide (PbI_2_, 98%), Guanidine Hydrobromide (GABr, 98%) were purchased from TCI (Tokyo, Japan). SnO_2_ colloid (15 wt%) was supplied by Alfa Aesar (Shanghai, China). N,N-dimethylformamide (DMF, >99%), dimethyl sulfoxide (DMSO, >99%), Isopropyl Alcohol (IPA, >99%), chlorobenzene (99%), Acetonitrile (>99%), (4-tert-Butylpyridine (tBP, >99%) and lithium-bis(trifluoromethanesulfonyl)imide (Li-TFSI, >99%) were purchased from Sigma-Aldrich (St. Louis, MO, USA). 2,2′,7,7′7-tetrakis-(N,N-di-p-methoxyphenylaminor)-9,9′-spiroobifluorene(Spiro-OMeTAD, >99.5%) and Methylamine Hydroiodide (MAI, 99.5%) were purchased from Xi’an polymer light Technology Crop (Xi’an, China).

### 2.2. Preparation of Devices

ITO coated glass substrates were cleaned by diluted detergent and rinsed with deionized water. Then, the substrates were cleaned by ultra-sonication with deionized water, alcohol for 15 min in sequence and dried by N_2_ gas. After drying, the substrates were sent to an ultraviolet ozone chamber and treated for 15 min. SnO_2_ precursor solution was prepared by mixing SnO_2_ colloid and deionized water (volume ratio, 1:3). SnO_2_ layer was spin-coated on the substrate at 4000 rpm for 20 s and then annealed at 160 ℃ for 20 min. After being cooling down, the ultraviolet-ozone treatment on SnO_2_ was carried out and the duration was set as 15 min. In this study, MAPbI_3_ is prepared by one-step spin-coating deposition method with anti-solvent and then GABr treatment is carried out and optimized by varying the concentrations of GABr in IPA. The perovskite precursor solution was prepared by dissolving 714.56 mg PbI_2_ and 246.38 mg MAI (molar ratio, 1:1) in 1 mL DMF/DMSO (volume ratio, 9:1). The MAPbI_3_ was spin-coated on SnO_2_ at 4000 rpm for 25 s, and the antisolvent of 200 μL chlorobenzene was dripped at 10 s in the process in N_2_-filled glove box. The as-prepared perovskite samples were sequentially annealed at 60 ℃ for 2 min and then 110 ℃ for 25 min. The GABr solutions were prepared by dissolving x mg GABr powder into 1 mL IPA corresponding to different concentrations. The GABr was then dropped on and spin-coated quickly at 4000 rpm for 30 s. Then the film was annealed at 105 ℃ for 5 min. Spiro-OMeTAD solution was prepared by dissolving 72.3 mg Spiro-OMeTAD, 17.5 mL Lithium salt solution (520 mg Li-TFSI in 1 mL Acetonitrile) and 28.75 mL tBP in 1 mL chlorobenzene. Spiro-OMeTAD was spin-coated at 3000 rpm for 30 s and then the samples were stored in a chamber filled with dried oxygen for 12 h. Afterward, Au electrode (~70 nm) was deposited by vacuum evaporation with a shadow mask. 

As for the devices for SCLC test, the SnO_2_ was substituted by PEDOT:PSS, and it was spin-coated on substrate at 5000 rpm for 30 s, and annealed at 150 ℃ for 15 min. Then, the substrates were sent to glove box directly for the next step without other treatment. 

The flexible devices were prepared in the same way except for substrates cleaning and annealing process of SnO_2_. The ITO coated PEN was used as received without cleaning. The SnO_2_ was annealed at 110 ℃ for 50 min.

### 2.3. The Characterization of Films and Devices

The XRD patterns were obtained by an X-ray diffractometer (XRD, Shimadzu XRD-6100, Tokoy, Japan) with Cu-Kα (λ = 1.5405 Å) radiation source. The absorption spectra were obtained by Lambda 950 UV/Vis spectrophotometer (PerkinElmer Inc., Waltham, MA, USA). The surface and cross-sectional images were observed by field-emission scanning electron microscopy (FE-SEM, Regulus-8230, Hitachi, Tokyo, Japan). The AFM images and KPFM images were obtained by using an atomic force microscopy (DI Multimode 8, Bruker Nano Inc., Munich, Germany). PL and TRPL spectra were measured by FLS980 fluorescence spectrometer (Edinburgh Inc., Livingston, Scotland, UK). XPS and UPS spectra were measured by using a photoelectron spectrometer (ESCALAB 250Xi, Thermo Fisher Scientific, Waltham, MA, USA). The *J-V* measurements and SCLS tests were conducted by using a Keithley 2634B source meter (Cleveland, OH, USA) under AM 1.5G illumination (100 mW cm^−2^). The active area of cells was defined by a shadow mask as 0.0975 cm^2^. The spectral response measurements were performed by a solar cell quantum efficiency measurement system (QEX10, PV Measurements, Inc., Boulder, CO, USA). 

## 3. Results and Discussion

**Figure 1:**
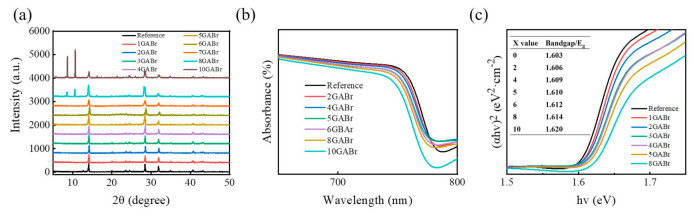
(**a**) XRD patterns of pristine MAPbI_3_ and perovskite films, (**b**) the UV-vis absorption spectra, and (**c**) (αhv)^2^ versus energy of perovskite films, treated by GABr with different concentrations.

X-ray diffraction (XRD) is carried out to explore the impact of different concentrations of GABr on the crystal structure of MAPbI_3_. Figure 1a shows the XRD patterns of perovskites treated with different concentrations of GABr. x GABr denotes x mg·ml^−1^ GABr treatment in this work unless otherwise noted. The diffraction peaks at 14.08°, 28.42° and 31.88° corresponding to the (110), (220), and (330) planes of MAPbI_3_, and the peak at 12.78° is assigned to PbI_2_, which disappears after GABr treatment [37,38]. The intensity of MAPbI_3_ peaks gradually increases at low concentrations of GABr, whereas when x is higher than 5, the intensity decreases. When x = 8, two new diffraction peaks at 8.71° and 10.72° appear, and this indicates the formation of 2D GA_2_PbI_4_ [39]. Furthermore, GA^+^ might locate at the grain boundary or the surface of the perovskite layer, rather than entering the lattice of MAPbI_3_ because there is no obvious shift observed in MAPbI_3_ peaks. Notably, with the increased concentration of GABr, the intensity of GA_2_PbI_4_ peaks increases while that of MAPbI_3_ decreases. This suggests that an appropriate content of GABr will enhance the crystallinity of MAPbI_3_, but excess GABr will disrupt the crystallinity and generate 2D GA_2_PbI_4_. Afterwards, we apply ultraviolet-visible (UV-vis) absorption spectroscopy to observe the alternation of optical properties of perovskite layers. The absorption spectra are shown in Figure 1b, and the absorption band edge shifts to short wavelength with the increased x. As MAPbI_3_ is a direct-bandgap semiconductor, the optical bandgap can be calculated by Tauc relationship illustrated in Equation (1):

(αhν)^2^ = C (hν−E_g_), (1)

where α is absorption coefficient, h is Planck’s constant, ν is frequency of vibration, and C is proportional constant. Then, we obtain the Tauc plot shown in Figure 1c, the optical bandgap (E_g_) is calculated from the intersection between the cut-off edge and the x-axis. E_g_ becomes larger with the increasing concentration of GABr, revealing the incorporation of Br into MAPbI_3_ lattice by partially replacing I site. 

**Figure 2:**
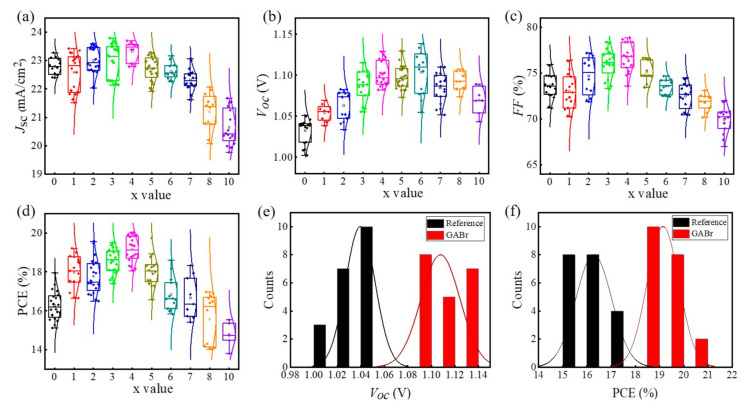
Statistical *J-V* parameters of (**a**) *J*_sc_, (**b**) *V*_oc_, (**c**) *FF*, and (**d**) PCE of devices modified by different concentrations of GABr. The histogram of (**e**) *V*_oc_ and (**f**) PCE for reference devices and GABr-treated devices.

The current density-voltage (*J-V*) measurement is carried out to directly understand the influence of GABr post-treatment on the device performance. The typical *J-V* parameters including short circuit current density (*J*_sc_), open circuit voltage (*V*_oc_), fill factor (*FF*), and PCE are summarized and depicted in Figure 2a–d. The results indicate that the GABr treatment induces a significantly improvement of *V*_oc_ when x < 7, and slightly decrease after x exceeds 7. *J*_sc_ and *FF* keep increasing as the concentration increases from 0 to 4, but when x ≥ 5, *J*_sc_ and *FF* drop severely. The devices achieve the best performance at x = 4 and give the highest *V*_oc_ = 1.148 V at x = 6. This indicates that moderate concentration of GABr can achieve an obvious promotion of device performance, while excess GABr will deteriorate the device performance. Herein, we denote 4 GABr as GABr in the following content, unless the special explanation. Then, we statistic the *V*_oc_ and PCE of devices without and with GABr treatment to evaluate the effect of GABr treatment (Figure 2e,f). The average *V*_oc_ of reference devices is 1.033 V, whereas the average *V*_oc_ of GABr-based devices is 1.108 V. The average PCE’s are 16.2% and 19.2% for pristine devices and treated devices, respectively.

Remarkably, the hysteresis effect tends to be mitigated when x < 5, but when x further increases, severe hysteresis is observed again. Hysteresis Index (HI) is one parameter commonly used to evaluate the hysteresis degree, and HI is defined as:

HI = (PCE_RS_−PCE_FS_)/PCE_RS_, (2)

in which the PCE_RS_ is the efficiency measured under reverse scan direction, and PCE_FS_ represents the efficiency measured under forward scan direction [40]. The HI values of different GABr-treated devices are summarized in Table S1 and the *J-V* curves are shown in Figure S1. Obviously, one can find that moderate GABr post-treatment can reduce the HI, but when the concentration of GABr keeps increasing, the hysteresis becomes critical. Actually, hysteresis issue occurs seriously in MAPbI_3_-based PSCs with n-i-p planar structure is associated with the dipole moment of MA cation [41]. The suppressed hysteresis effect should be affected by the nearly zero dipole moment of GA^+^ [25]. Besides, GA^+^ tends to form hydrogen bonds with the unsaturated defects center, which can powerfully suppress ion migration and mitigate the hysteresis effect [19].

**Figure 3:**
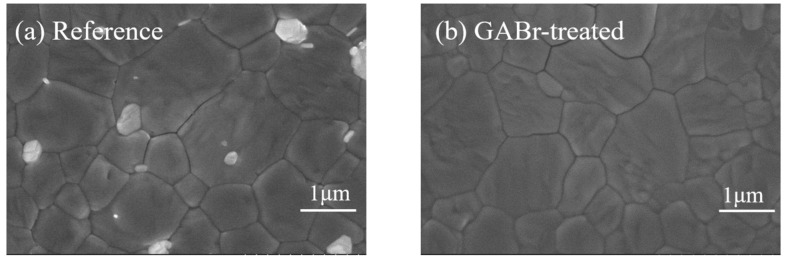
SEM images of (**a**) the reference film, (**b**) GABr-treated MAPbI_3_.

The surface morphology of perovskite films is shown in Figure 3. One may find that there are some bright particles on the surface of the pristine MAPbI_3_, which might be indexable to the excess PbI_2_ [42,43]. After treatment, these particles disappear and maintain a dense and pinhole-free surface. When the concentration of GABr increases to x = 8 mg·mL^−1^, we suppose that excess GABr will react with the PbI_2_ inside the perovskite lattice combined with XRD spectra, and then forming a two-dimensional GA_2_PbI_4_ capping layer with disordered and smaller crystals (as shown in Figure S2). Due to the insulating nature of 2D perovskite, this capping layer is supposed to hinder the carrier transportation and cause performance degradation [30].

**Figure 4:**
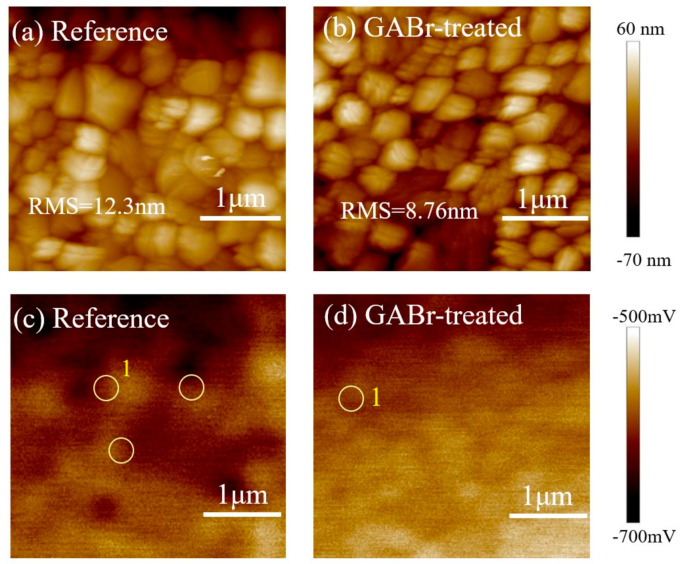
AFM morphology of (**a**) the reference film, (**b**) GABr-treated MAPbI_3_. KPFM images of (**c**) the reference film and (**d**) GABr-treated MAPbI_3_. In Figure 4c,d, the yellow circle represents the type of grain boundary #1.

According to the AFM profile of the pristine and GABr-treated MAPbI_3_ film shown in Figure 4a,b, GABr-treated film shows RMS = 8.76 nm, which is slightly lower than that of the pristine one (RMS = 12.3 nm), which is consistent with the SEM results. The decreased surface roughness is beneficial for the device performance because it can effectively reduce the contact resistance between perovskite layer and HTL. Moreover, we conduct Kelvin probe force microscopy (KPFM) to observe the surface potential change after GABr treatment as shown in Figure 4c,d. GABr-treated film presents a more uniform surface potential than reference film. It has been demonstrated that the grain boundaries surrounded by grains with large contact potential difference (illustrated as #1 in Figure 4c,d) would induce severe hysteresis in local photocurrents [44]. The decrease in the number of grain boundaries #1 in GABr-treated film suggests that GABr treatment is beneficial to produce superior perovskite with more uniform surface potential and thus forcefully suppress the hysteresis effect. 

**Figure 5:**
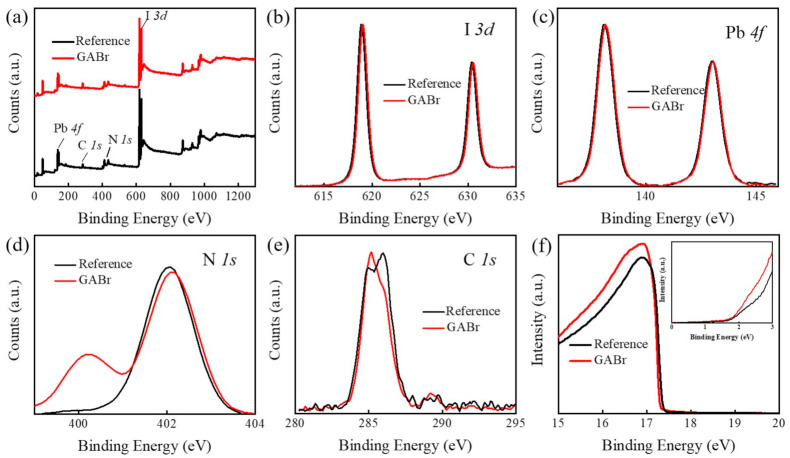
(**a**) The elemental survey spectra of MAPbI_3_ and GABr-treated MAPbI_3_. High-resolution XPS spectra of (**b**) I 3d, (**c**) Pb 4f, (**d**) N 1s, (**e**) C 1s of MAPbI_3_ and GABr-treated MAPbI_3_. (**f**) The UPS secondary electron edge cut-offs of these two samples, and the inner image is the corresponding magnified details of the valence band spectra.

The influence in chemical component of GABr treatment is investigated by X-ray photoelectron spectroscopy (XPS). Figure 5b–e show the core spectra of I 3d, Pb 4f, N 1s, and C 1s. The spectra of N 1s can be divided into two peaks in GABr-treated samples: the peak at 402.04 eV attributed to NH_3_^+^ group in MA, and the peak at 400.12 eV to NH_2_^+^ group in GA [45]. The spectra of I 3d, N 1s and Pb 4f exhibit a slight shift after post-treatment, which indicates that GABr treatment will induce an influence on the chemical environment of these elements. The ratio of each element is listed in Table S2, the Pb/I ratio decreases from 39.3% to 37.5% after GABr treatment, which further confirms the reaction between PbI_2_ and GABr.

Furthermore, the work function (W_F_) and the valence band (VB) maximum are measured by ultraviolet photoelectron spectroscopy (UPS). Figure 5f shows the secondary electron edge cut-offs (E_cutoff_) and valence band maximum (E_VBM_) spectra of pristine and GABr-treated MAPbI_3_. The E_cutoff_ and E_VBM_ are obtained by the extrapolation of the line fit to the cut-off edge. According to Einstein’s photoemission law (W_F_ = hν − E_cutoff_ − E_F_), W_F_ is calculated to be 3.837 eV and 3.917 eV, and the E_VBM_ is obtained to be 1.47 eV and 1.45 eV of MAPbI_3_ and GABr-treated sample [46]. This result reveals that the film treated by GABr is less n-type, and an upward shift of E_VBM_, which will decrease the charge transport barrier between perovskite and Spiro-OMeTAD. The modified band alignment can make more effective charge separation and collection, which might effectively suppress the charge recombination at the interface and improve the device performance [47,48].

**Figure 6:**
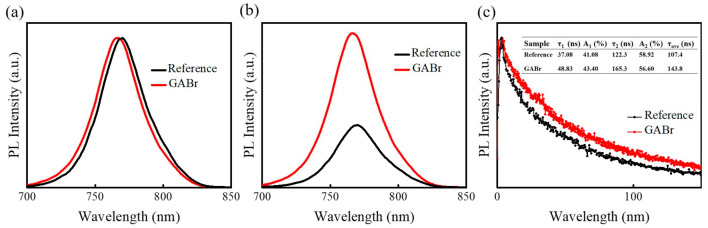
(**a**) The normalized Photoluminescence (PL) spectra of MAPbI_3_ and GABr-treated film. (**b**) PL and (**c**) time-resolved PL (TRPL) results of MAPbI_3_ and GABr-treated films, and the inset table is the corresponding parameters of the fitting results.

Afterwards, pristine MAPbI_3_ and GABr-treated perovskite film are prepared on quartz substrate for steady-state Photoluminescence (PL) and time-resolved PL decay (TRPL) measurement. In Figure 6a, the PL emission peak shifts from 770 nm to 766 nm after GABr treatment, which is in keeping with the absorption spectra. Moreover, the intensity of PL peak of GABr-treated film is much stronger than the pristine film, suggesting a superior film with fewer density of defects and suppressed nonradiative recombination (as shown in Figure 6b) [49,50,51]. The TRPL curve can be fitted by a biexponential equation [23]:

f(t) = A_1_ exp(−t/τ_1_) + A_2_ exp(−t/τ_2_), (3)

where τ_1_, τ_2_ refer to the fast-decay and long-decay recombination mechanism, A_1_, A_2_ are the corresponding coefficients. The fast decay is related with the rapid charge transfer at interlayer, and the long process is attributed to the bulk and interface recombination [52]. After calculation, GABr-treated MAPbI_3_ shows both increasement in τ_1_ and τ_2_ (the fitting results are given in the table inserted in Figure 5c). The average carrier lifetime (τ_ave_) is estimated according to the following equation [53]:

τ_ave_ = (A_1_ τ_1_^2^ + A_2_ τ_2_^2^)/(A_1_ τ_1_ + A_2_ τ_2_), (4)

The τ_ave_’s are 107.4 ns and 143.8 ns for the reference perovskite and GABr-treated film, respectively. The longer decay time of GABr-treated film further indicates fewer defect states and a suppression of nonradiative recombination [54]. It is well known that GA^+^ cation is a Lewis acid with a symmetrical structure, which allows the formation of hydrogen bonds between the partial negative (δ^−^) iodine ions and partly positively charged (δ^+^) ammonium H atoms [19,55]. Overall, we propose that after post-treatment, GA^+^ trends to bond with free iodide ion and thus successfully passivates the nonradiative recombination.

**Figure 7:**
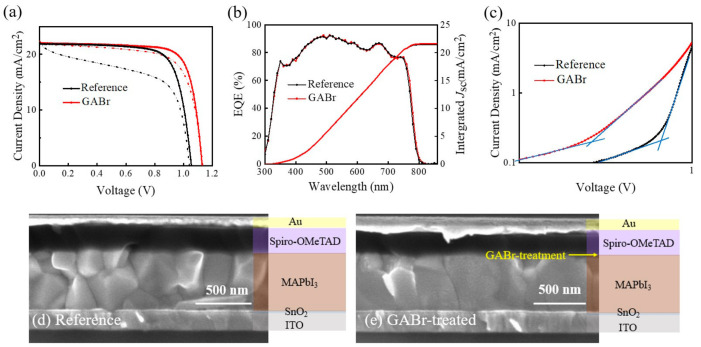
(**a**) The *J-V* curves, (**b**) external quantum efficiency (EQE) spectra and (**c**) space-charge-limited current (SCLC) results of the champion devices based on MAPbI_3_ and GABr-treated device. The cross-sectional SEM images of (**d**) reference devices, and (**e**) GABr-based devices.

The *J-V* curves of the champion devices are displayed in Figure 7a. The pristine device achieves a best PCE of 17.95% with a *V*_oc_ of 1.055 V, a *J*_sc_ of 22.93 mA/cm^2^ and an *FF* of 74.21%, and the GABr-treated device shows better performance with overall improvement in *V*_oc_ (1.13 V) and *FF* (76.81%) while maintaining the performance of *J*_sc_ (23.06 mA/cm^2^). The *J-V* parameters of devices measured under different scan directions are summarized in Table S3. Figure 7b shows the external quantum efficiency (EQE) spectra of the champion performance devices. The integrated *J*_sc_’s are 21.49 mA/cm^2^ for reference device and 21.63 mA/cm^2^ for the GABr-treated device, which is consistent with *J-V* measurement result. 

To further understand the passivation effect of GABr, a space-charge-limited current (SCLC) measurement is conducted to calculate the density of defect. Then, we prepare devices with structure of ITO/PEDOT:PSS/PVSK/Spiro-OMeTAD/Au to investigate the trap-state density (n_trap_). The n_trap_ can be obtained according to equation [23]:

n_trap_ = 2εε_0_V_TFL_/qL^2^, (5)

where ε is the relative dielectric constant of the perovskite material, ε_0_ is the vacuum permittivity, L is the thickness of perovskite layer, q is the electron charge and V_TFL_ is the trap-filled limit voltage [56,57]. The calculated n_trap_’s are 8.1 × 10^15^ cm^−3^ and 2.04 × 10^16^ cm^−3^ for GABr-treated MAPbI_3_ and pristine MAPbI_3_, respectively. The decreased trap-state density after GABr treatment means lower charge recombination rate and improved film quality, which might lead to higher *V*_oc_ and *FF* of device [53,58,59]. 

Devices with a structure of ITO/SnO_2_/MAPbI_3_/Spiro-OMeTAD/Au are made for cross-sectional SEM characterization. As shown in Figure 7d,e, compared with the reference perovskite layer composed of grains with various sizes, GABr-treated film displays fewer grain boundaries and more uniform grain size. The interface between MAPbI_3_ and Spiro-OMeTAD is more uniform after GABr treatment, which suggests a lower contact resistance. The reduction of grain boundaries should be helpful to suppress the ion diffusion between the perovskite layer and transport layer of the devices [60]. Meanwhile, GABr-treated perovskite performs almost vertical grain boundaries, which attesting the decreasing of carrier trap interface and photocurrent conduction pathway [61].

**Figure 8:**
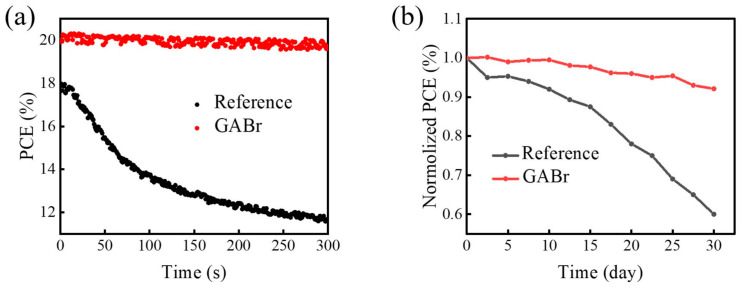
(**a**) Steady-state power conversion efficiency (PCE) of the champion devices with/without treatment, (**b**) Long-term stability trace of champion devices stored and tested in N_2_-filled glove box after 30 days.

Moreover, we monitor the stability performance of reference and GABr-treated devices (shown in Figure 8). GABr-treated device shows a steady-state PCE of 19.95% in a duration of 300 s, while untreated device experiences serious decay. The poor steady-state PCE of untreated device should be resulted from the aggravated ion migration due to the increased temperature under continuous illumination, and serious nonradiative recombination induced by defects [5,62]. The long-term stability is also recorded (the devices are stored and tested in a N_2_-filled glove box) and the GABr modified device can retain over 90% of its original PCE after 30 days. On the contrary, the efficiency of the controlled one rapidly degraded to 90% of the initial value in merely 10 days. The main reason of the optimized stability for GABr treated devices is the improved crystallinity with decreased defect states of the perovskite layer, which enhances its resistance to moisture, oxygen and/or light irradiation [27,55].

Thus, we have proved that GABr treatment can result in a substantial improvement in the performance of MAPbI_3_ PSCs fabricated on rigid glass. Then we attempt to verify the effect of GABr treatment on flexible devices. The XRD and SEM spectra of perovskite with/without GABr treatment are shown in Figures S3 and S4. Interestingly, the perovskite films show preferred orientation. After modification, the continuity of the film is improved while the grain boundary gets blurred. The unusual crystal orientation and surface morphology should be caused by the special properties of the underneath substrates. 

**Figure 9:**
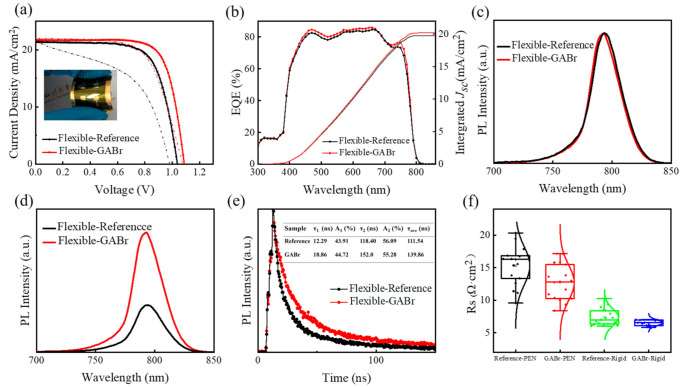
(**a**) The *J-V* curves and (**b**) EQE spectra of the champion cells based on MAPbI_3_ and GABr-treated flexible device. (**c**) The normalized PL spectra of MAPbI_3_ and GABr-treated film. (**d**) PL and (**e**) TRPL results of MAPbI_3_ and GABr-treated films, and the inset table is the corresponding parameters of the fitting results. (**f**) The statistical results of R_s_ of pristine devices and GABr-based devices deposited on two substrates.

The *J-V* curve and EQE spectra of the champion devices based on MAPbI_3_ and GABr-treated perovskite are shown in Figure 9a,b. On a flexible substrate, GABr-devices still exhibit a significant enhancement in *V*_oc_ with slight improved *J_s_*_c_ and *FF* and achieve a best PCE of 17.57%. The integrated *J*_sc_’sare 19.93 mA/cm^2^ and 20.34 mA/cm^2^ of pristine and GABr-treated device, respectively. Compared with rigid devices, the EQE response in wavelength region of 300–400 nm is suppressed in flexible devices due to the strong absorption of PEN-ITO in this region (the transmittance spectra of the two substrates are shown in Figure S5). HI index values and *J-V* parameters are listed on Table S4. The HI effect is also being reduced after GABr treatment. The PL and TRPL spectra of flexible samples are shown in Figure 9c–e. The tendency is consistent with the samples deposited on rigid substrates. The carrier lifetime is prolonged to 139.85 ns after post-treatment, while the lifetime of pristine film is 111.54 ns.

The main issues of the performance of flexible devices are the low *FF* due to the high series resistance (R_s_), and the worse *J*_sc_ affected by the surface properties the flexible substrate [63]. R_s_ of the controlled and GABr-treated devices deposited on different substrates is summarized in Figure 9f. This result indicates that GABr post-treatment can effectively reduce the R_s_ of the devices, which can be attributed to the modified surface morphology of perovskite layer [33]. The obstacles in the development of flexible PSCs based on PEN-ITO or PET-ITO are the worse efficiency, brittleness of ITO and the relatively high cost. It still needs more efforts to exploit cheaper conductive electrode materials with better bending resistance and transparency and employ effective strategies to boost the application. 

## 4. Conclusions

In conclusion, we propose a simple GABr-post treatment to enhance the crystallinity of MAPbI_3_, increase the carrier lifetime, and boost the PCE to over 20% on rigid substrate. The average *V*_oc_ is increased by 80 mV and the serious hysteresis in MAPbI_3_-based planar devices is significantly eliminated after GABr treatment. The application of GABr leads to a better band energy matching between perovskite and spiro-OMeTAD, which is beneficial for charge transport. In addition, the treatment results in a superior MAPbI_3_ film and can effectively passivate the trap states. This work also demonstrates that GABr treatment is a feasible approach to improve the efficiency of MAPbI_3_ solar cells fabricated on flexible PEN substrate. Thanks to the eliminated *V*_oc_ loss after GABr treatment, the PCE of flexible devices increases from 15.77% to over 17.57%. 

## Data Availability

The data presented in this study are available on request from the corresponding author.

## Supplementary Materials

The following are available online at https://www.mdpi.com/2079-4991/11/3/750/s1, Figure S1: *J-V* curves of devices treated by high concentration of GABr measured under different scan directions, Figure S2: (a) SEM image of 8 mg·mL^−1^ GABr treated MAPbI_3_ and (b) the cross-sectional SEM image of 8 mg·mL^−1^ GABr treated solar cell device, Figure S3: XRD spectra of MAPbI_3_ and GABr treated MAPbI_3_ deposited on flexible substrates, Figure S4: SEM images of (a) MAPbI_3_ and (b) GABr treated MAPbI_3_ deposited on flexible substrates, Figure S5: The transmittance spectra for both substrates for rigid and flexible devices, Table S1:HI values of devices treated by different concentrations of GABr, Table S2: Atomic ratio of MAPbI_3_ and GABr-treated film obtained from XPS data, Table S3: *J-V* parameters of the champion devices based on MAPbI_3_ and GABr-treated MAPbI_3_ on rigid substrates, Table S4: *J-V* parameters and HI values of the champion devices based on MAPbI_3_ and GABr-treated MAPbI_3_ on flexible substrates.
