# Supplementary material for: GABr Post-Treatment for High-Performance MAPbI3 Solar Cells on Rigid Glass and Flexible Substrate
Source: Nanomaterials (Basel). 2021 Mar 16;11(3):750. doi: 10.3390/nano11030750 (PMC8002339; doi:10.3390/nano11030750)
Supplement: Supplementary file 1 [file nanomaterials-11-00750-s001.pdf]

nanomaterials

Supplementary Materials

GABr Post-Treatment for High-Performance $MAPbI_3$ Solar Cells on Rigid Glass and Flexible Substrate

Tingting Chen [1], Rui He [1], Fan Zhang [2], Xia Hao [2,3,*] , Zhipeng Xuan [2], Yunfan Wang [2], Wenwu Wang [1,3], Dewei Zhao [1,3], Jingquan Zhang [1,3] and Lili Wu [1,3,*]

1 College of Materials Science and Engineering, Sichuan University, Chengdu 610065, China; chentingting1@stu.scu.edu.cn (T.C.); hrkbjj@stu.scu.edu.cn (R.H.); www1492@scu.edu.cn (W.W.); dewei.zhao@scu.edu.cn (D.Z.); zhangjq@scu.edu.cn (J.Z.)
2 Institute of New Energy and Low-Carbon Technology, Sichuan University, Chengdu 610065, China; zhangfan3@stu.scu.edu.cn (F.Z.); 2018226220003@stu.scu.edu.cn (Z.X.); wangyunfan@stu.scu.edu.cn (Y.W.)
3 Engineering Research Center of Alternative Energy Materials & Devices, Ministry of Education, Chengdu 610065, China
* Correspondence: hao.xia0808@scu.edu.cn, wulili@scu.edu.cn

Citation: Chen, T.; He, R.; Zhang, F.; Hao, X.; Xuan, Z.; Wang, Y.; Wang, W.; Zhao, D.; Zhang, J.; Wu, L.; et al. GABr Post-Treatment for High-Performance $MAPbI_3$ Solar Cells on Rigid Glass and Flexible Substrate. *Nanomaterials* **2021**, *11*, 750. https://doi.org/10.3390/nano11030750

Academic Editor: Jung-Ho Yun

Received: 1 February 2021
Accepted: 11 March 2021
Published: 16 March 2021

Publisher's Note: MDPI stays neutral with regard to jurisdictional claims in published maps and institutional affiliations.

Copyright: © 2021 by the authors. Licensee MDPI, Basel, Switzerland. This article is an open access article distributed under the terms and conditions of the Creative Commons Attribution (CC BY) license (http://creativecommons.org/licenses/by/4.0/).

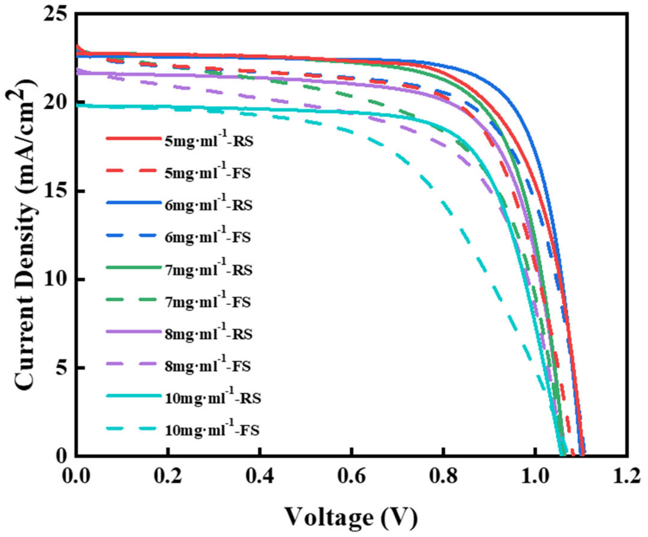

Figure S1. *J-V* curves of devices treated by high concentration of GABr measured under different scan directions.

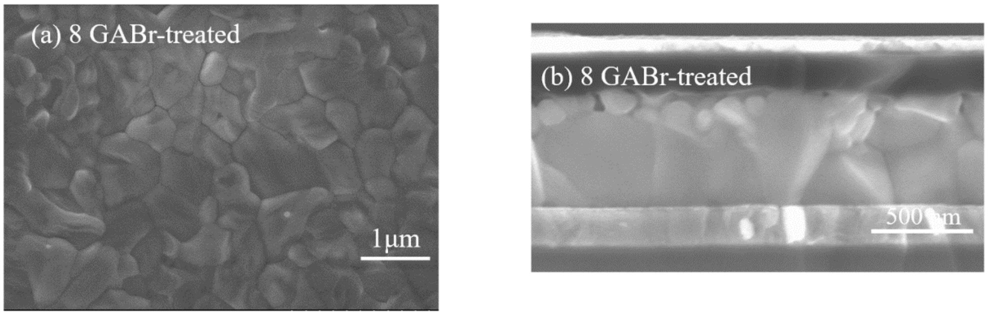

Figure S2. (**a**) SEM image of 8 mg·ml^{-1} GABr treated $MAPbI_3$ and (**b**) the cross-sectional SEM image of 8 mg·mL^{-1} GABr treated solar cell device.

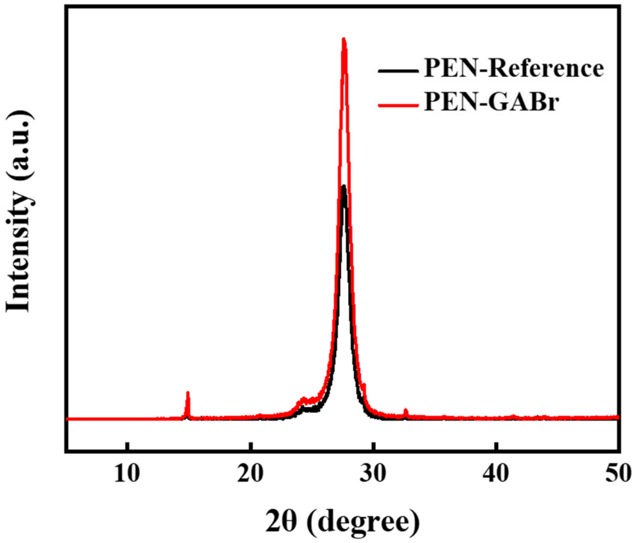

Figure S3. XRD spectra of $MAPbI_3$ and GABr treated $MAPbI_3$ deposited on flexible substrates.

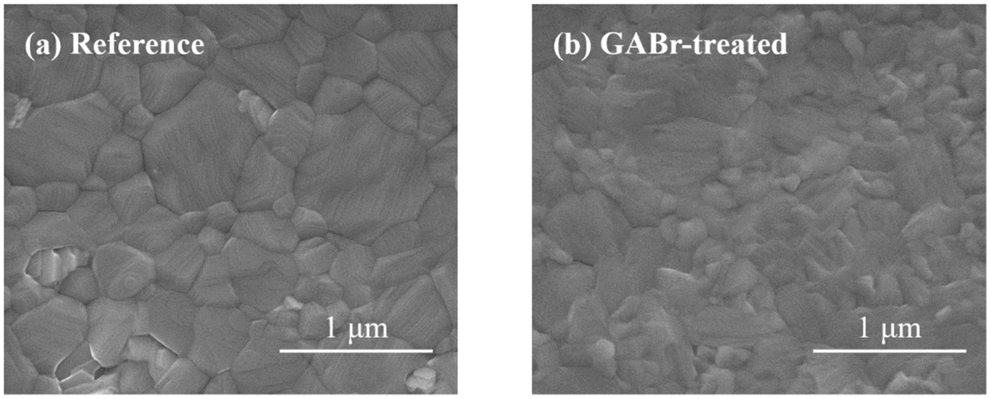

Figure S4. SEM images of (**a**) $MAPbI_3$ and (**b**) GABr treated $MAPbI_3$ deposited on flexible substrates.

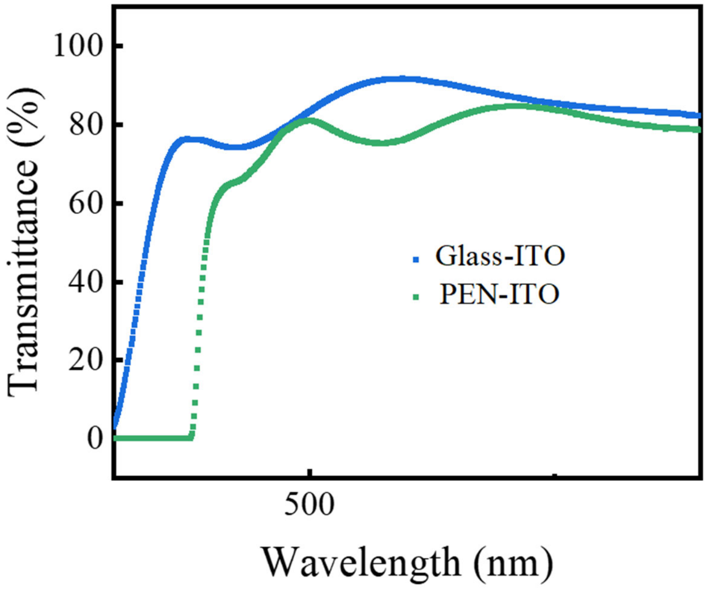

Figure S5. The transmittance spectra for both substrates for rigid and flexible devices.

Table S1. HI values of devices treated by different concentrations of GABr.

**x value**	0	1	2	3	4	5	6	7	8	10
**HI (%)**	30.49	29.9	11.08	5.25	2.46	13.29	17.36	19.63	19.19	28.42

Table S2. Atomic ratio of $MAPbI_3$ and GABr-treated film obtained from XPS data.

**Elements**	**Atomic Ratio of Elements (%)**	
	**Reference**	**GABr**
I 3d	66.69	68.34
Pb 4f	26.18	25.65
C 1s	5.58	3.37
N 1s	1.55	2.50
Br 3d	0	0.14

Table S3. *J-V* parameters of the champion devices based on $MAPbI_3$ and GABr-treated $MAPbI_3$ on rigid substrates.

**Sample**	**J_{sc} (mA/cm^2)**	**V_{OC} (V)**	**FF (%)**	**PCE (%)**
reference-forward	23.26	1.041	56.24	13.62
reference-reverse	22.93	1.055	74.21	17.95
GABr-forward	23.39	1.132	69.03	18.27
GABr-reverse	23.06	1.130	76.81	20.01

Table S4. *J-V* parameters and HI values of the champion devices based on $MAPbI_3$ and GABr-treated $MAPbI_3$ on flexible substrates.

**Sample**	**J_{sc} (mA/cm^2)**	**V_{OC} (V)**	**FF (%)**	**PCE (%)**	**HI (%)**
reference-forward	22.11	1.041	49.36	11.36	27.96
reference-reverse	21.29	1.055	70.21	15.77	
GABr-forward	22.0	1.078	64.43	15.28	13.03
GABr-reverse	21.71	1.09	74.25	17.57	
